# Automatic bridge crack detections: A lightweight segmentation model for UAV inspection

**DOI:** 10.1371/journal.pone.0358865

**Published:** 2026-09-24

**Authors:** Xuwei Dong, Zhibing Yu, Jiashuo Yuan, Jinpeng Dai

**Affiliations:** 1 Key Laboratory of Opto-Electronic Technology and Intelligent Control, Ministry of Education, Lanzhou Jiaotong University, Lanzhou, China; 2 School of Electronics and Information Engineering, Lanzhou Jiaotong University, Lanzhou, China; 3 National and Provincial Joint Engineering Laboratory of Road & Bridge Disaster Prevention and Control, Lanzhou Jiaotong University, Lanzhou, China; 4 School of Materials Science and Engineering, Southeast University, Nanjing, China; TU Dublin Blanchardstown Campus: Technological University Dublin - Blanchardstown Campus, IRELAND

## Abstract

Bridge cracks are among the primary factors affecting structural safety and durability. Intelligent and precise detection and quantification of cracks are important in improving bridge operation and maintenance efficiency and safety assessment. Traditional manual detection methods are inefficient and highly subjective, while existing deep learning methods still have shortcomings in terms of computational overhead and detailed modelling. Therefore, this study proposes a high-precision and lightweight crack semantic segmentation network—Feather SFASwinUnet. The model introduces two new modules: Scale Fusion Attention (SFA) and Feather ConvBlock (FCB). SFA effectively fuses global semantic information and local detailed features through a dual-branch structure, which improves spatial structure modelling capabilities. Meanwhile, FCB compensates for feature distortion with extremely low overhead in skip connections, which enhances the recovery capability of fine-grained cracks. Experimental results show that, on the public dataset SDNET2018, the proposed model achieves an mIoU value of 81.25% and an mDice value of 88.57%. It significantly outperforms typical methods such as Unet, DeepLabV3+ and SwinUnet while maintaining low computational load. Furthermore, the YOLO11-BD detection algorithm is combined to achieve crack detection and segmentation under complex backgrounds, which effectively improves robustness. Lastly, the orthogonal skeleton method is used to quantitatively analyse the segmentation results. As a result, the measurement errors of crack width and length are controlled within 5%, which provides reliable technical support for bridge structural health assessment and maintenance decisions. The Feather SFASwinUnet proposed in this study provides an efficient and scalable technical approach for automatic crack detection and quantitative analysis of bridges under UAV inspection. In addition, it has high engineering application potential.

## 1 Introduction

Bridges as one of the earliest infrastructures used to optimise traffic conditions, play an important role in the development of human society [1]. In modern urban road management, bridges are key engineering facilities for improving space utilisation efficiency and travel efficiency. Bridges are also among the costliest types of infrastructure because of their complex design and difficult construction [2]. By the end of 2024, the total number of highway bridges in China had reached 1.11 million, and the role of bridges in the national transportation system has become increasingly prominent. However, bridges will develop cracks due to load changes and corrosion during long-term service, which will affect structural performance and accelerate steel corrosion and concrete deterioration. If not repaired in time, then they may cause serious damage and endanger traffic safety [3,4]. Therefore, long-term health monitoring and regular inspection of bridges are crucial to ensuring their safe and stable operation throughout their entire life cycle [5,6]. Cracks are one of the most common structural defects of bridges and an early signal of surface deterioration. If they can be detected and repaired in time, then they will not only help significantly reduce maintenance costs but also effectively prevent potential catastrophic consequences [7]. However, early bridge crack detection mainly relied on manual visual inspection and traditional measuring tools, which are cumbersome and highly subjective; thus, inconsistencies exist in bridge condition assessments across different inspectors or regions [8,9].

With the continuous advancement of computer science, various computer vision algorithms have been proposed and widely applied in different fields. At the same time, the development of unmanned aerial vehicle (UAV) has also been used to improve the bridge detection capability in dangerous areas for addressing the limitations of traditional bridge crack detection methods [10]. In terms of bridge crack detection, existing models can be mainly divided into three categories: crack detection models based on traditional image processing methods, machine learning and deep learning [11]. Traditional image processing techniques mainly include edge detection [12], morphological operations [13], threshold segmentation [14], region growing [15] and template matching [16]. Nguyen et al. [17] proposed to use the geometric characteristics of cracks in 2D images that are ‘dendritic’ and ‘centrally symmetric’ to realise crack edge detection and distinguish cracks from the edges of other objects. Dorafshan et al. [18] proposed an automatic concrete surface crack detection method combining Otsu threshold segmentation and morphological operations. This method can effectively distinguish cracks from noise and achieve efficient detection without training data and dye penetration. Thus, it is suitable for UAV inspection images. Song et al. [19] proposed a photovoltaic image crack detection algorithm based on multi-scale pyramid and improved region growing. This algorithm effectively suppresses noise, removes suspected cracks and completes cracks. It has high detection accuracy and is suitable for solar cell defect identification in complex backgrounds. Although digital image processing technology has improved the recognition efficiency in crack detection, it is more sensitive to background noise, which limits its detection accuracy and adaptability. This problem has prompted researchers to explore more accurate intelligent methods, such as machine learning technology, to improve the reliability and practicality of crack recognition [20]. Gavilan et al. [21] proposed an automatic road crack detection system based on SVM with road surface classification adaptive adjustment parameters. This approach effectively improves the crack recognition accuracy on different types of road surfaces and reduces false and missed detection. Prasanna et al. [22] proposed a crack detection method called Spatial Tuned Robust Multi-Feature, which uses machine learning (SVM, Adaboost and RF) to classify crack and non-crack pixels. The accuracy of the results is significantly higher than that of traditional image processing methods. Shi et al. [23] proposed the CrackForest method, which uses random structure forest and structured features to realise automatic road crack detection, effectively identify complex topological cracks and significantly suppress noise. Thus, this method improves the accuracy and efficiency of crack detection. Traditional machine learning methods can achieve relatively ideal detection results under certain specific conditions, but they have difficulty achieving robust crack recognition in variable environments due to their limitations in feature extraction capabilities. By contrast, in recent years, an important branch of machine learning—deep learning technology—has shown excellent performance in crack image detection tasks [24]. The rise of deep learning has greatly promoted the widespread application of convolutional neural networks in computer vision tasks such as image classification, object detection and image segmentation. By constructing multi-layered neural network structures, deep learning can automatically learn layered feature representations from large-scale datasets, which effectively improves the crack detection capability of the model in complex scenarios [25,26]. The mainstream target detection networks (e.g., You Only Look Once, YOLO) [27] and Single Shot MultiBox Detector, SSD [28]) have also been applied to crack classification and localisation tasks. By generating bounding boxes, they accurately depict the contours of cracks, which demonstrates their adaptability and detection efficiency on various surface types [29]. Liu et al. [30] proposed a rotating target detection method based on improved YOLOv5 by introducing angle regression, attention mechanism and feature fusion module. This method is mainly used for high-efficiency identification of cracks in concrete bridges. Zou et al. [31] proposed a bridge crack detection method YOLOv8s-MA based on improved YOLOv8s. This method introduces the C2f-MA attention module and a small target detection layer, which effectively improve the detection accuracy of small cracks in complex backgrounds. Dong et al. [32] proposed a lightweight bridge crack detection algorithm YOLO11-BD based on improved YOLO11. This algorithm uses the EMSCA module and LDH detection head, which significantly reduce the amount of computation while improving detection accuracy and achieving efficient real-time detection.

Although these methods have shown high performance in crack identification, classification and target detection, their analytical capabilities are limited to identifying cracks as a whole target because convolutional neural networks mainly process at the image or target level. Moreover, they have difficulty providing fine information regarding the spatial distribution of cracks. These limitations restrict the capability to accurately assess the integrity of crack structural features (e.g., shape, size and distribution) [33,34]. In recent years, semantic segmentation models have been widely used to extend crack analysis to the pixel level, which has promoted the development of various end-to-end methods for pixel-level crack detection [35,36]. Encoder–decoder network structures (e.g., DeepLab [37], SegNet [38] and Unet [39]) have been widely used for semantic segmentation of cracks. They extract global contextual information and fine-grained features from crack images and aggregate them into a compact expression; then, they decode and reconstruct them into high-precision pixel-level segmented images [40]. Fu et al. [41] proposed an improved bridge crack semantic segmentation method based on the Dense-DeepLabv3 + network. They changed the ASPP module to a dense connection structure, enhanced the feature extraction capability, increased the pixel sampling density and receptive field and improved the segmentation accuracy while keeping the number of parameters unchanged. Pang et al. [42] proposed an improved SegNet bridge crack semantic segmentation method. By reducing the network depth, introducing the ConvNeXtV2 module and improving the loss function, they enhanced the accuracy and accelerated the detection speed. Su et al. [43] proposed an improved U-Net bridge crack recognition method CBAM-Unet based on the CBAM attention mechanism. Combined with image post-processing technology, they achieved accurate crack segmentation and effectively improved detection accuracy and efficiency. Although U-Net has achieved good results in bridge crack segmentation, U-Net essentially relies on the local convolution operation of CNN; thus, it has difficulty fully modelling long-distance dependencies and global context information [44]. Under this background, the Transformer architecture has made breakthroughs in the field of natural language processing with its self-attention mechanism and has gradually expanded to computer vision tasks. In particular, the proposal of Vision Transformer [45] and its subsequent improvement Swin Transformer [46] has provided a new modelling paradigm for image segmentation, which enables the combination of local and global information. Qi et al. [47] proposed a new network VCV-Net that combines Vision Transformer and variational level set theory to achieve fine segmentation of bridge deck cracks and other defects. This method significantly improves segmentation accuracy. Wang et al. [48] proposed the SwinCrack network by integrating Swin Transformer and convolutional modules to achieve continuous and accurate detection of long and thin cracks. They verified this network on multiple public datasets. To integrate the structural advantages of U-Net and the modelling capabilities of Swin Transformer, Cao et al. [49] proposed the SwinUnet model. For the first time, they constructed a U-shaped network based on a pure Transformer architecture. SwinUnet exhibits excellent segmentation accuracy and strong generalisation capability. It opens up a new direction for the development of bridge crack segmentation. Cabral et al. proposed a hybrid pipeline combining YOLO11 with the Segment Anything Model (SAM) for multi-damage segmentation in UAV inspection imagery, achieving promising results on cracks, efflorescences, and exposed rebars [50].

In recent years, numerous methods based on CNNs, Transformers and their fusion architectures have emerged in the semantic segmentation of bridge cracks. These studies have significantly reduced the number of model parameters and improved the capability to capture local details by introducing lightweight convolutions and attention mechanisms [51,52]. Furthermore, they have enhanced the overall accuracy of crack segmentation through global context modelling. However, all these methods lack a truly practical framework that can simultaneously retain the inherent advantages of the Unet series in multi-scale feature extraction and fully integrate global semantic information to achieve end-to-end comprehensive modelling for bridge crack semantic segmentation. In the field of automated bridge crack detection, high-resolution images of the bridge structure are currently typically captured by drones at a safe distance. The resulting massive amounts of data significantly increase the computational cost of subsequent segmentation models. To address these issues, this study proposes a segmentation network model—Feather SFASwinUnet, which combines Feather ConvBlock (FCB) and Scale Fusion Attention (SFA) to enhance SwinUnet. Firstly, the proposed method designs a dual-branch parallel architecture SFA to fuse global and local crack image information and dynamically adjusts the weight ratio to improve the spatial attention capability of the model. Then, in the skip fusion channel of the decoding stage of SwinUnet, FCB is designed to compensate for feature distortion caused by dimension alignment in the Skip-Connection channel with extremely low computational overhead, which can significantly reduce inference latency and memory usage when processing drone images. Next, the effectiveness and advantages of the proposed segmentation model are verified on the public dataset SDNET2018. At the same time, combined with the YOLO11-BD algorithm proposed in our previous study, the detection and segmentation of bridge cracks under complex backgrounds are realised [32]. Lastly, the orthogonal skeleton method is used to quantify the segmentation results and accurately calculate the length and width of bridge cracks. Thus, this work provides reliable technology support for bridge structural health assessment and maintenance decisions.

## 2 Methodology

### 2.1 Feather SFASwinUnet

In processing the task of crack segmentation from drone images, the model needs to effectively fuse multi-scale information while maintaining the resolution of the feature map to accurately capture tiny cracks and their edge details [53]. Given that the traditional Swin Transformer uses a fixed window size, the sliding of its window position on the image will cause discontinuity between regions, which limits its capability to express the image. In addition, SwinUnet has significant storage and computing overhead when processing inputs such as 4K level. The video memory requirement can reach 16 GB to 24 GB, and the inference time also increases significantly. Meanwhile, the Transformer architecture has inherent limitations in modelling high-frequency information. Especially when faced with fine texture structures (e.g., crack edges), key details are often lost due to insufficient feature expression capabilities. This limitation is mainly due to that its processing of high-frequency information relies on shallow nonlinear transformations and lacks an effective high-frequency feature enhancement mechanism, which results in obvious shortcomings in the microstructure recovery of the model.

**Fig 1:**
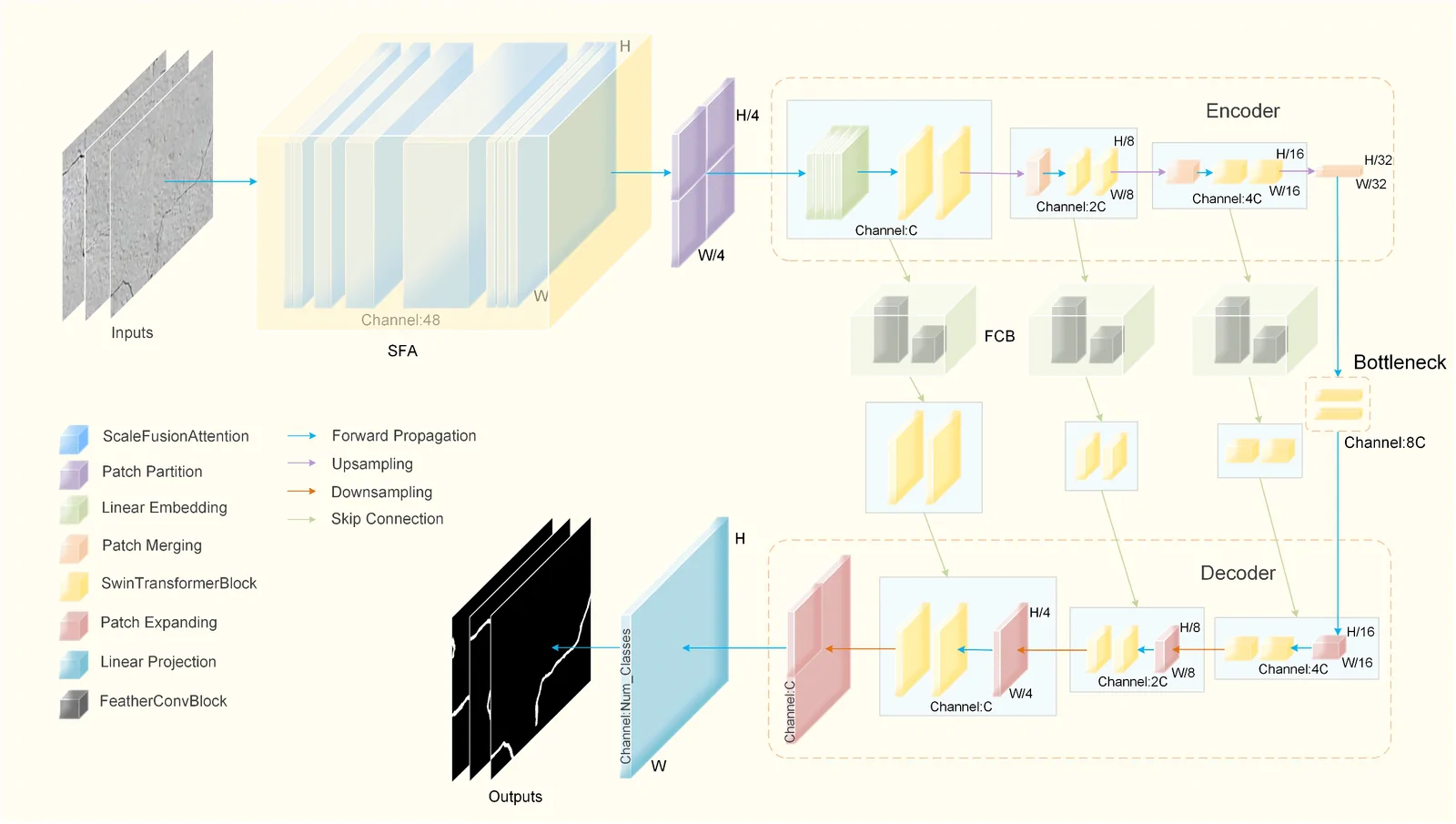
Architecture of the feather SFASwinUnet network.

The overall structure of Feather SFASwinUnet, which is a deep neural network architecture for crack segmentation proposed in this study, is shown in Fig 1. It mainly consists of an encoder, a bottleneck module and a decoder. The input image is processed by shallow convolutional encoding to generate an initial feature map. This map is then weighted and fused using the SFA module to achieve multi-scale spatial feature fusion. The SFA is placed before Patch Partition and reconstructs the crack region spatially through a multi-scale perception mechanism. This mechanism guides the subsequent Transformer encoder to perform more effective self-attention modelling of salient regions. In the encoder part, the network adopts a hierarchical Swin Transformer structure, which utilises Patch Merging and Shifted Window Attention to achieve progressive resolution compression and long-range dependency modelling. Each encoding module consists of a standard Swin Transformer Block and extracts progressively enhanced semantic feature representations through linear projection and hierarchical feature transfer. The bottleneck layer, which is located in the deepest feature space, is responsible for fusing global contextual semantics and providing high-level feature guidance to the decoder. Subsequently, the decoder restores spatial resolution through progressive Patch Expanding operations. At the same time, it introduces skip connections to fuse shallow high-resolution features from the encoder for maintaining the integrity of edge information. To improve detail preservation during upsampling, FCB are embedded at the skip connections of each layer in the decoding module. The lightweight convolutional structure and local detail enhancement mechanism employed by FCBs collaboratively reconstruct shallow and deep features. This feature effectively improves the shortcomings of traditional Transformers in high-frequency feature restoration.

#### 2.1.1 Scale fusion attention.

**Fig 2:**
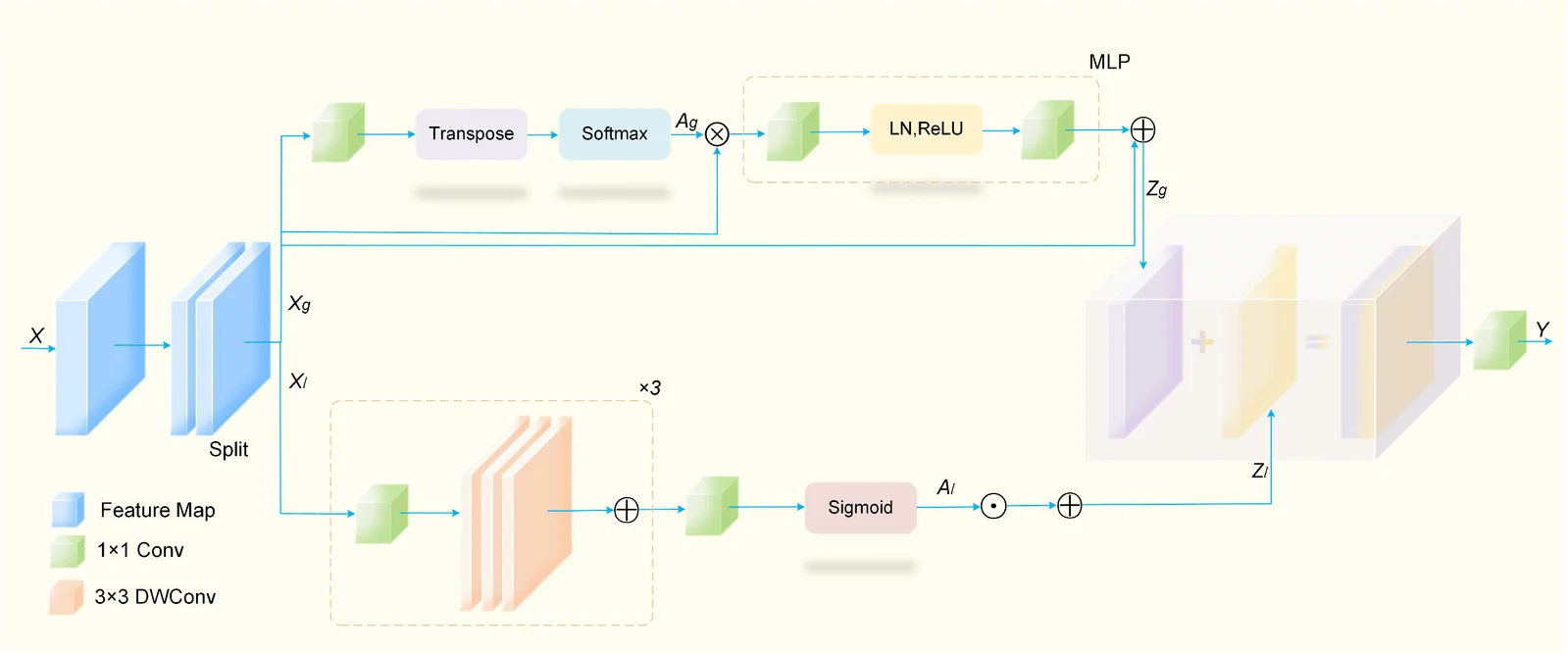
Architecture of the SFA network.

Traditional attention mechanisms often struggle to simultaneously model global dependencies and preserve local details, particularly when modelling high-density boundary targets such as cracks and contours. Furthermore, semantic features at different scales exhibit significant distributional differences in spatial alignment and semantic consistency, which easily lead to insufficient information fusion. To enhance the fine-grained understanding of target edges, structural information and contextual relationships in multi-scale visual scenes by the model, this study designs an attention module, SFA, with semantic consistency reconstruction and structural awareness capabilities. This module aims to collaboratively integrate global context and local structural information, which achieves efficient fusion within the feature domain through a dual-branch architecture. The structure of the SFA model is shown in Fig 2.

SFA includes a global attention branch and a local attention branch, which are respectively responsible for modelling long-range dependencies and maintaining high-frequency details. SFA performs channel grouping processing on the input feature map, and it aggregates information under receptive fields of different scales through dynamic selection mechanisms (e.g., channel division and weighted fusion). SFA divides the input feature map $X\in R^{B\times H\times W\times C}$ into $X_{g}$ and $X_{l}$ according to the channel dimension. Then, it sends them to the global attention branch and the local attention branch, as shown in Equation (1):


$$\begin{array}{c} X_{g},\text{\textbackslash{}hspace\{0.33em}\}X_{l}=Split\left(X\right) \end{array}$$ (1)

The global branch uses a simplified global context encoder to capture long-range dependencies and enhance the non-local correlation between pixels in the image, such as Equations (2) and (3):


$$\begin{array}{c} A_{g}=Softmax\left(Transpose\left(C_{1\times 1}\left(X_{g}\right)\right)\right) \end{array}$$ (2)

$$\begin{array}{c} Z_{g}=MLP(A_{g}⊗X_{g})+X_{g} \end{array}$$ (3)

Softmax is a normalisation function, which makes the sum of attention weights equal to 1 on the channel or space to highlight the salient areas. Transpose is the transpose operation used to adjust the dimension of the attention weight. ⊗ represents matrix multiplication. MLP represents a two-layer fully connected layer with activation function and normalisation. The local branch uses lightweight convolution to enhance the expression of spatial edge details. It focuses on strengthening the spatial characteristics of small areas, such as edges or small target areas, as shown in Equations (4) and (5):


$$\begin{array}{c} A_{l}=\sigma \left(C_{1\times 1}\left(F_{c}\left(X_{l}\right)\right)+X_{l}\right) \end{array}$$ (4)

$$\begin{array}{c} Z_{l}=A_{l}⊙X_{l}+X_{l} \end{array}$$ (5)

$F_{c}(\cdot )$ represents a submodule consisting of three 1 × 1 convolutions and a 3 × 3 depth convolution; σ is the Sigmoid function, and ⊙ is point-wise multiplication. Lastly, 1 × 1 convolution is used to fuse the two, and the most discriminative features are further screened out through the attention gating mechanism. This procedure achieves effective reconstruction of cross-scale semantic information, as shown in Equation (6):


$$\begin{array}{c} Y=C_{1\times 1}\left(Concat\left(Z_{g},\text{\textbackslash{}hspace\{0.33em}\}Z_{l}\right)\right) \end{array}$$ (6)

Compared with traditional attention mechanisms, SFA considers global semantic understanding and local structure recovery capabilities while maintaining computational efficiency. It is especially suitable for segmentation tasks of fine-grained targets such as cracks. As a general attention component, this module can be flexibly embedded in the network and significantly improve the perception accuracy of the model for targets with blurred edges and fine structures.

#### 2.1.2 Feather ConvBlock.

In a typical encoder–decoder structure, the decoding stage often fuses the shallow spatial features of the encoder and the deep semantic features of the decoder through skip connections. However, due to the significant difference between semantic level and spatial resolution, direct splicing often leads to inconsistent fusion feature expression and weakened edge response, especially in fine-grained target segmentation such as cracks. To effectively improve the context expression capability and local crack texture identification capability after multi-scale feature fusion, this study proposes a lightweight and functionally enhanced feature modelling module called FCB. This module is integrated into the Skip-Connection channel at each stage of the decoder. It uses an efficient convolution structure to reconstruct the structure sensitivity of the fused features, which alleviates the problems of semantic drift and boundary blur after cross-scale splicing. The design of FCB is based on the concept of depthwise separable convolution, which decouples traditional convolution operations into two independent stages to model spatial information and channel interaction, respectively. This way significantly reduces the number of parameters and improves feature decoupling capabilities.

**Fig 3:**
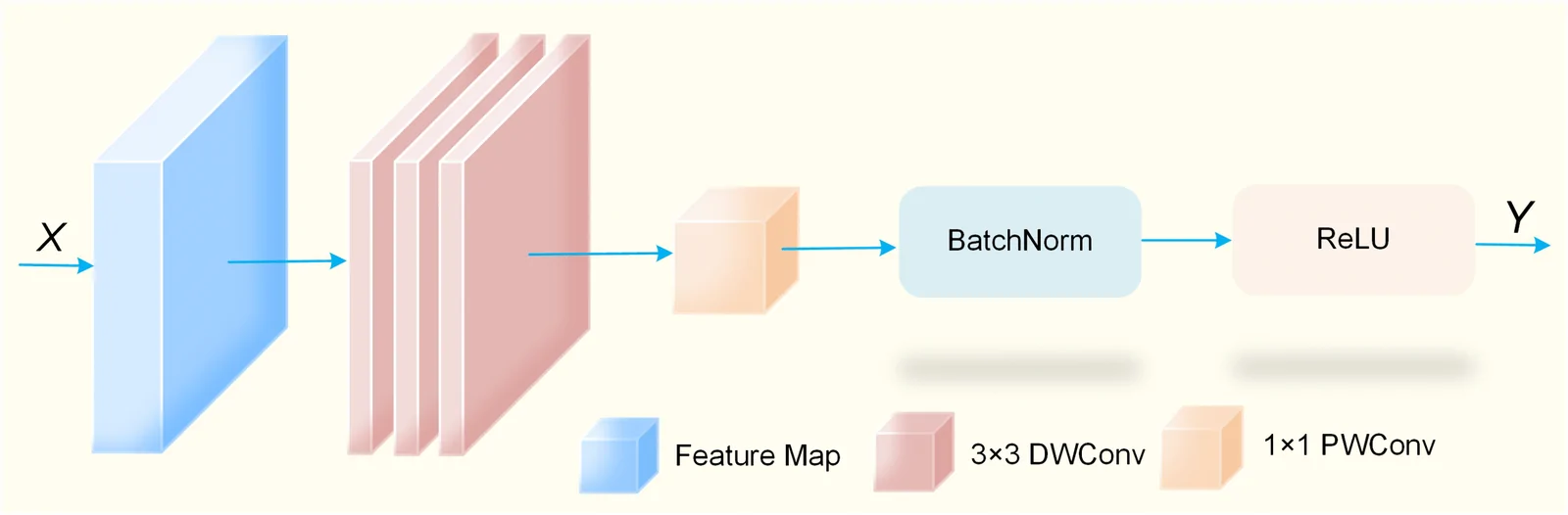
Architecture of the FCB network.


The structure of FCB is shown in Fig 3. Given the input feature map $X\in R^{B\times H\times W\times C}$, the forward propagation process of FCB is shown in Equation (7):

$$\begin{array}{c} Y=ReLU\left(BN\left(PWConv_{1\times 1}\left(DWConv_{3\times 3}\left(X\right)\right)\right)\right) \end{array}$$ (7)

$DWConv_{3\times 3}$ performs spatial convolution independently by channel and explicitly models the local response pattern of each channel, $PWConv_{1\times 1}$ is used for information reconstruction and fusion of channel dimensions, BN is utilised to alleviate internal covariate offsets and improve convergence stability and ReLU introduces nonlinear enhancement capabilities.

Such a design only contains k^2^ × C + C^2^ convolution kernels, which compensates the feature distortion caused by dimensional alignment in Skip-Connection with extremely low overhead. The amount of parameters and calculations is low, which can significantly reduce the inference delay and memory usage. Such low amount is particularly friendly to edge deployments.

### 2.2 Orthogonal skeleton method

The orthogonal skeleton method is a morphological representation method based on geometric and topological features. Its core idea is to extract a skeleton that can maintain the overall topological structure and simplify morphological features by constructing orthogonal equidistant lines of the target boundary. Firstly, the algorithm calculates the orthogonal distance distribution from its boundary point to the nearest boundary based on the target contour. Then, it forms a skeleton curve by finding the contraction and intersection positions of these equidistant lines. This skeleton curve not only can truly reflect the global geometric characteristics of the original shape of targets such as cracks but also can eliminate the interference of local noise at the boundary to a large extent.

**Fig 4:**
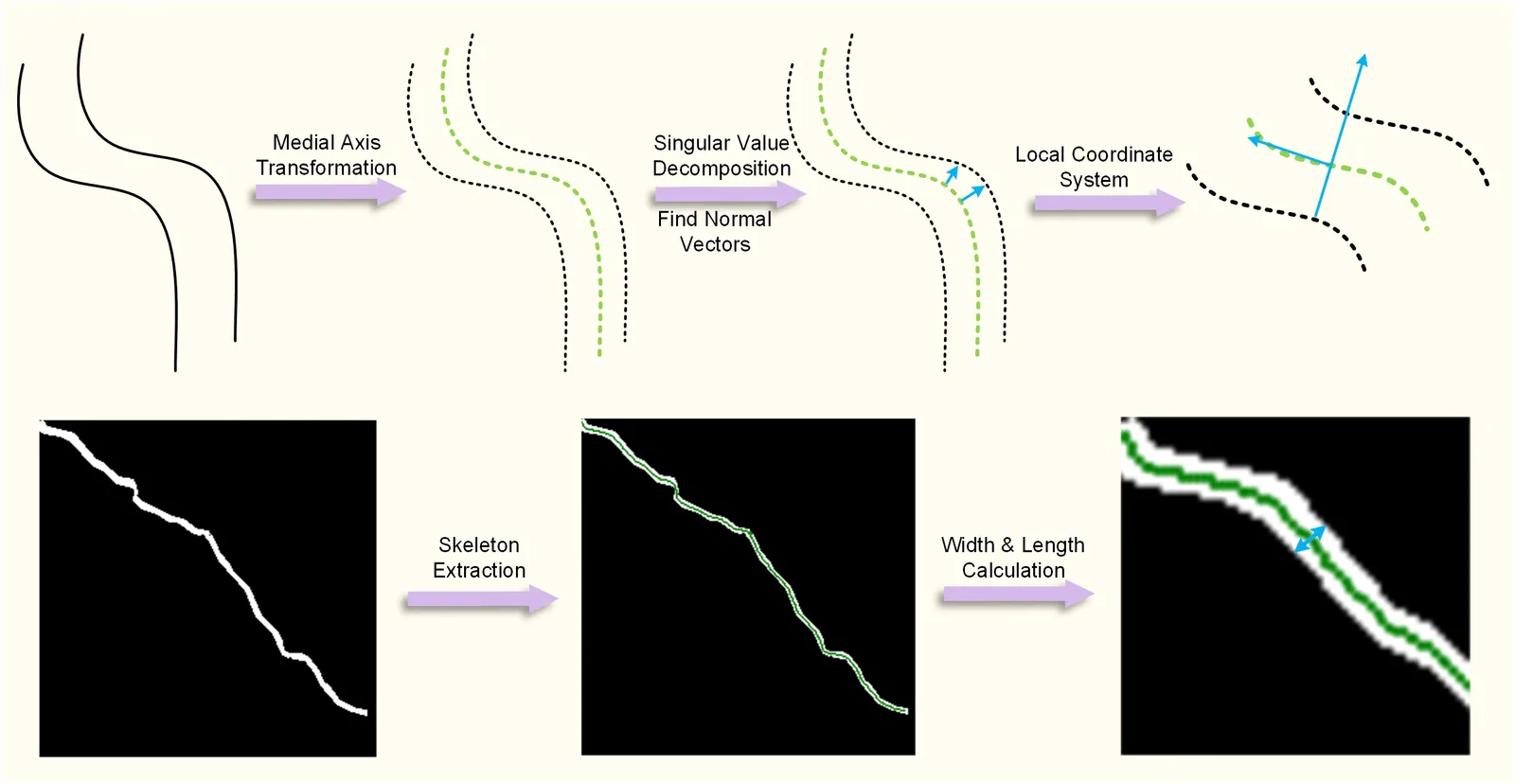
Orthogonal skeleton method.

As shown in Fig 4, for the crack width measurement at a specified location, the algorithm first constructs a kd-tree based on the k-nearest neighbour points of the location to improve the efficiency of neighbourhood search. Then, singular value decomposition is used to calculate the local normal vector of the skeleton line, which is used as the y-axis direction of the local coordinate system. Next, the x-axis direction orthogonal to it is determined. By projecting the crack edge line into the local coordinate system, the two intersection points of the skeleton normal vector and the edge line can be effectively found. Notably, the distance between the two intersection points is used as a measure of the local crack width. Similarly, after the skeleton line is extracted, the length of the crack can be obtained by calculating the total number of all pixels on the skeleton path.

## 3 Experimental process and result analysis

### 3.1 Dataset of SDNET2018

This study uses the publicly available dataset SDNET2018 to train and evaluate the proposed method while ensuring the reproducibility of the study and the comparability of the results. The dataset was constructed by researchers at Utah State University and covers more than 56,000 images of concrete cracks and non-cracks, including multiple concrete structural parts such as bridge decks, walls and sidewalks. The crack width in the image ranges from 0.06 mm to 25 mm, and the crack contains a variety of complex interference factors such as shadows, surface roughness, scaling changes, edge interference, holes and background impurities. In this study, the subset containing bridge deck images with cracks is selected. This part of the data comes from the Utah State University system, material and structural health (SMASH) laboratory. It contains a total of 2,025 images with a resolution of 256 × 256. Given that the crack labelling information is not provided in this dataset, this study uses the Labelme tool for manual labelling and generates corresponding mask maps. Lastly, the dataset is divided into training, validation and test sets in a ratio of 8:1:1.

### 3.2 Experiment configuration

All experimental procedures are carried out on a dedicated computational server. The hardware and software specifications are provided in Table 1 for reproducibility.

**Table 1:** Experimental hardware and software configurations.

Component	Model/ Specification	Key Parameters
CPU	Intel Xeon Platinum 8352V	16 vCPUs, base frequency 2.10 GHz
GPU	NVIDIA GeForce RTX 4090	24 GB GDDR6X VRAM
Memory	DDR4 RAM	64 GB, 3200 MHz
Storage	NVMe SSD (Gen4)	1 TB, high-speed data I/O
Operating System	Ubuntu	22.04 LTS
Deep Learning Framework	PyTorch	2.3.0, with CUDA 12.1

The choice of hyperparameters also plays an important role in the performance of the model. For calculation efficiency and GPU memory limitations, the batch size is set to 16. The training epoch is set to 100 rounds. The Cosine Annealing Learning Rate Scheduler is used to prevent the learning rate from declining too fast in the late training period and affecting the model training results. The scheduler gradually reduces the learning rate throughout the training process. The initial learning rate is set to 0.01, the minimum value is 1e-5, the total scheduling period is the same as the total training epoch and the learning rate is adjusted according to the following Equation (8):


$$\begin{array}{c} \eta_{t}=\eta_{min}+\frac{\text{1}}{\text{2}}(\eta_{\text{0}}-\eta_{min})\left(1+cos\left(\frac{t}{T_{max}}\pi \right)\right) \end{array}$$ (8)

where $\eta_{t}$ represents the current learning rate of the t-th epoch, $\eta_{\text{0}}$ represents the initial learning rate, $\eta_{min}$ refers to the minimum learning rate, which is the lower limit of the learning rate attenuation and $T_{max}$ is the total training epoch.

For the loss function, a combined loss composed of CrossEntropyLoss and DiceLoss is adopted with a weight ratio of 0.4:0.6. This combination is chosen to mitigate the severe class imbalance inherent in crack segmentation, where crack pixels account for only a small proportion of all pixels. DiceLoss directly optimizes the overlap between the predicted segmentation and the ground truth, demonstrating robustness against class imbalance, while CrossEntropyLoss provides pixel-wise supervision to ensure stable convergence during training.

### 3.3 Evaluation indicators

In this experiment, Mean Intersection over Union (mIoU) [54] and Mean Dice Coefficient (mDice) [55] are used as the main evaluation indicators of semantic segmentation performance. The calculation equation are as shown in Equations (9) and (10):


$$\begin{array}{c} mIoU=\frac{\text{1}}{k+1}\sum_{i=0}^{k}\frac{p_{ii}}{\sum_{j=0}^{k}p_{ij}+\sum_{j=0}^{k}p_{ji}-p_{ii}} \end{array}$$ (9)

$$\begin{array}{c} mDice=\frac{\text{1}}{k+1}\sum_{i=0}^{k}\frac{2\times p_{ii}}{\sum_{j=0}^{k}p_{ij}+\sum_{j=0}^{k}p_{ji}} \end{array}$$ (10)

where k represents the total number of categories, $p_{ij}$ denotes the probability of predicting the true category i as category j and $p_{ii}$ is the probability of correctly predicting category i. The two indicators are widely used in natural image and medical image segmentation tasks and can more comprehensively measure the segmentation accuracy of the model. At the same time, Params is used to evaluate the amount of trainable parameters in the model for reflecting its complexity. Moreover, FLOPs are used to evaluate the amount of floating-point operations required in the model inference process for measuring the computing requirements.

### 3.4 Ablation experiments

To conduct comprehensive ablation analysis, this study adopts the original SwinUnet as the baseline model (denoted as “Basis” in all ablation tables).

*1) SFA:* To verify the effectiveness of SFA, this experiment designs a comparative experiment including five groups of different models, as shown in Table 2. Firstly, the experiment selects two attention mechanism modules, namely, CBAM and SE, and uses them to enhance the baseline model (Basis) for comparison. To further explore the performance of SFA at different locations in the network, two embedding strategies are designed in the experiment: one is to embed SFA into the final stage of the downsampling step, and the other is to embed SFA before the Patch Partition for performance comparison analysis. Experimental results show that the introduction of SFA significantly improves the performance of the baseline model. In particular, it achieves higher mIoU and mDice values than the traditional CBAM and SE attention mechanisms. Especially, when SFA is placed before the Patch Partition, the number of parameters of this model is the same as that of the baseline model. Moreover, it is much lower than the number of parameters of the model embedded in the downsampling position. This design makes the model more conducive to capturing finer local structural features. The mIoU value reaches 80.53%, and the mDice value reaches 88.03%, which demonstrate the potential of SFA in handling bridge crack image segmentation tasks.

**Table 2:** Comparison results of different models in the SFA ablation experiment.

Settings	Params(M)	FLOPs(G)	mIoU(%)	mDice (%)
Basis	27.15	5.92	77.07	85.31
+ CBAM Attention	28.40	5.92	77.89	86.20
+ SE Attention	28.40	5.92	78.23	86.72
+ SFA_down-sampling_	53.47	5.92	79.71	87.41
+ SFA_Patch Partition_	27.15	5.92	80.53	88.03

*2) FCB:* To verify the rationality and effectiveness of the proposed FCB design at Skip-Connection, FCB is compared with three common improvement strategies (Cross-Attention, Learnable Weighted Fusion and Attention Gate) on the dataset. Table 3 shows the comparison of results for each model on Parameters, FLOPs, mIoU and mDice indicators. The proposed FCB achieves the highest segmentation performance with only a slight increase in parameters, with mIoU reaching 80.72% and mDice reaching 88.18%. These values are better than those of other comparison strategies. This result verifies that FCB can effectively alleviate cross-scale semantic mismatch and boundary blur problems during the Skip-Connection process through lightweight convolution decomposition and structure-sensitive reconstruction. Thus, it improves the recovery capability of fine-grained structures such as cracks. FCB not only maintains low computational costs but also achieves excellent pixel-level crack segmentation accuracy. Therefore, it has great potential and practicality for application in actual UAV inspection scenarios.

**Table 3:** Comparison results of different models in the FCB ablation experiment.

Settings	Params(M)	FLOPs(G)	mIoU(%)	mDice (%)
Basis	27.15	5.92	77.07	85.31
+ Cross-Attention	27.54	6.09	79.02	86.88
+ Learnable Weighted Fusion	27.15	5.92	69.33	78.77
+ Attention Gate	27.34	6.00	80.17	87.76
+ FCB	27.35	6.01	80.72	88.18

*3) Joint Ablation Experiment:* This study also conducts a joint ablation experiment to further verify the collaborative enhancement effect of the proposed SFA and FCB in bridge crack image segmentation. Specifically, the changes in model performance under different combination strategies are compared. The experimental results are shown in Table 4. The results show that, after introducing the SFA and FCB modules simultaneously, the model reaches the best level in various indicators, with mIoU increasing to 81.25% and mDice reaching 88.57%. Therefore, the two modules are highly complementary and synergistic in crack image segmentation. Meanwhile, although the parameter amount increases slightly from 27.15 M to 27.35 M and FLOPs rise to 6.01 G after fusing the two modules, the values are still within the lightweight deployable range. The growth of its computing resource costs is controllable, and it has good practicality. The experimental results fully verify the complementary advantages of SFA in enhancing spatial structure understanding before encoding and FCB in recovering high-frequency details during decoding. This verification further confirms the rationality and effectiveness of the proposed Feather SFASwinUnet structural design.

**Table 4:** Comparison results of different models in the joint ablation experiment.

Settings	Params(M)	FLOPs(G)	mIoU(%)	mDICE(%)
Basis	27.15	5.92	77.07	85.31
+ SFA	27.15	5.92	80.53	88.03
+ FCB	27.35	6.01	80.72	88.18
+ SFA & FCB	27.35	6.01	81.25	88.57

### 3.5 Comparison with typical segmentation algorithms

**Fig 5:**
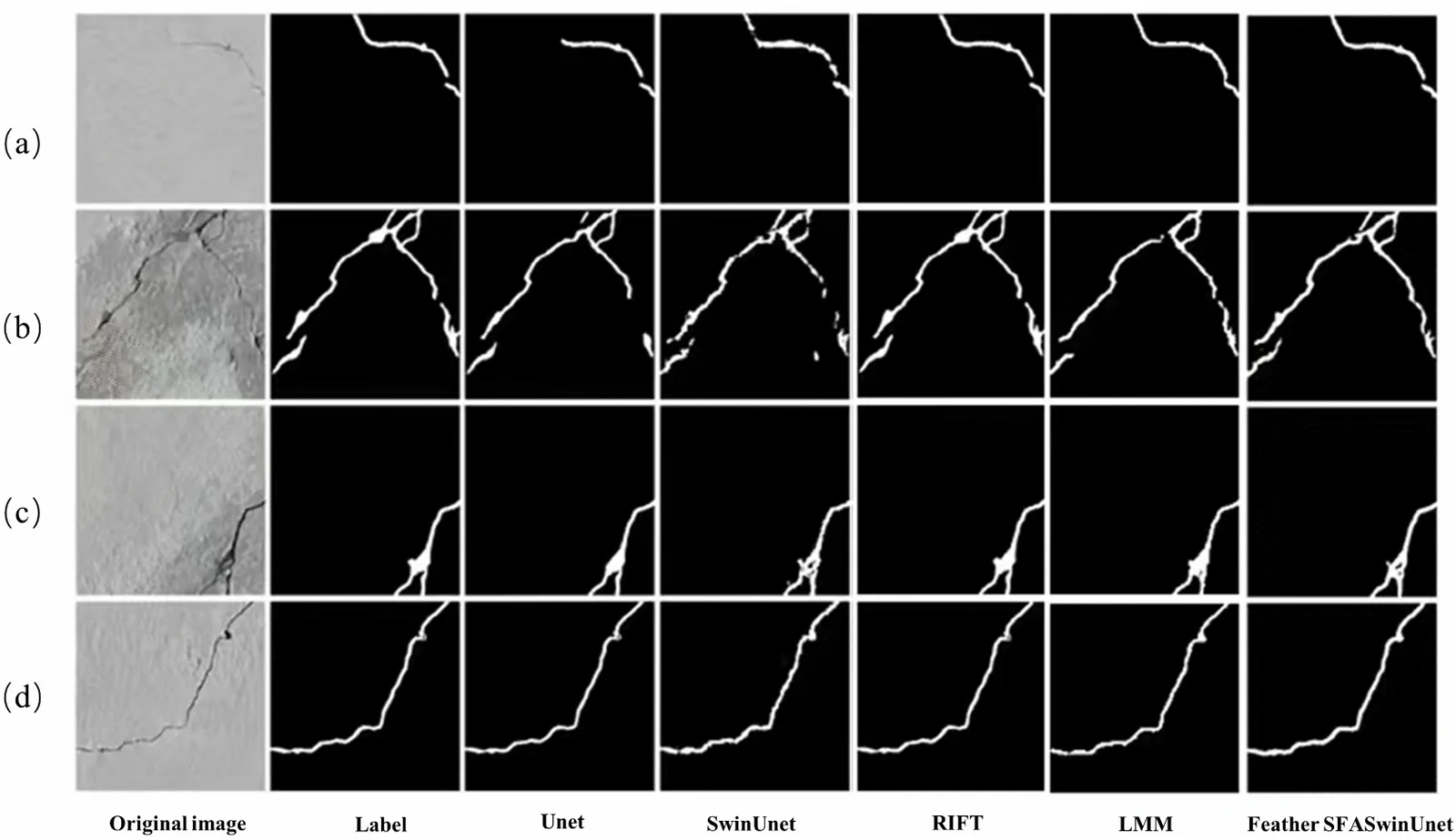
Comparison of inference results of different segmentation models.

To verify the superiority of Feather SFASwinUnet in the bridge crack image segmentation task, the proposed model is compared with different segmentation algorithms. Several representative semantic segmentation networks are selected, including Unet, DeepLabV3 + , SwinUnet, RIFT and LMM. These models are evaluated through experimental results on the dataset. In particular, their performance in terms of segmentation accuracy and computational costs is compared. Table 5 lists the performance of these models on the dataset. As shown in the table, Feather SFASwinUnet performs well in segmentation accuracy, with mIoU reaching 81.25% and mDice 88.57%. Compared with other methods, Feather SFASwinUnet is not only significantly better than Unet and SwinUnet in segmentation accuracy but also has a relatively small increase in computational costs.While lightweight models such as RIFT and LMM achieve low computational costs, their segmentation accuracy is far inferior to that of Feather SFASwinUnet.Therefore, it achieves a good balance between efficient calculation and high-precision segmentation. Fig 5 shows a comparison of model inference of different algorithms, which further confirms the advantages of Feather SFASwinUnet in segmenting small crack areas. Especially, when dealing with complex backgrounds and crack details, it can capture crack areas more accurately than other models. Although Unet has a low model complexity, its segmentation results are easily limited by the processing of edge details, and a certain degree of missed detection is observed. By contrast, Feather SFASwinUnet can effectively segment small crack areas. The effect is especially evident when dealing with edge blur and small crack intervals, which demonstrates its superior robustness. For example, Fig 5(a) and 5(b) show that the inference results of Feather SFASwinUnet even correct the errors of manually labelled ground-truth masks, including multi-labelled small fragment crack areas and small pothole crack areas.

**Table 5:** Comparison results of different segmentation models.

Settings	Params(M)	FLOPs(G)	mIoU(%)	FPS	mDice (%)
Unet	17.30	30.68	65.60	524.08	79.20
DeepLabV3+	39.60	27.45	74.80	182.06	83.45
SwinUnet	27.15	5.92	77.07	80.77	85.31
RIFT	1.6	0.63	74.36	164.00	79.58
LMM	0.82	2.07	65.78	48.46	76.54
Feather SFASwinUnet	27.35	6.01	81.25	86.20	88.57

### 3.6 Validation experiments on different datasets

To further explore the cross-domain generalization capability of the segmentation models, all comparison networks are trained and tested on the UAV-PDD2023 aerial dataset.

The SDNET2018 dataset consists of close-range crack images collected under well-controlled condition. In contrast, UAV-PDD2023 dataset contains outdoor aerial pavement images captured by unmanned aerial vehicles in complex field environments. The UAV-PDD2023 dataset covers multiple typical pavement crack categories, including longitudinal, transverse and reticulated cracks. Aerial imaging introduces inherent interferences such as motion blur, perspective distortion and uneven ambient light, which closely resemble the practical application scenarios of UAV-based bridge inspection. Table 6 summarizes all cross-domain experimental results. All models exhibit varying degrees of performance degradation when generalized from SDNET2018 to UAV-PDD2023. Conventional convolutional networks including U-Net and DeepLabV3 + suffer severe accuracy degradation. The lightweight models RIFT and LMM experience the most dramatic performance decline, while the original SwinUnet also exhibits considerable accuracy loss. The proposed Feather SFA SwinUnet achieves an mIoU of 80.25% and an mDice of 87.78% on UAV-PDD2023 dataset, with only 1.00% drop in mIoU and 0.79% drop in mDice compared with its performance on SDNET2018. Such marginal performance drop indicates that the embedded SFA and FCB modules facilitate the extraction of domain-invariant crack features, endowing the model superior cross-domain generalization capacity for UAV-based bridge crack detection. Furthermore, the UAV-PDD2023 dataset compensates the limitation of SDNET2018, which only consists of laboratory scenes. On this basis, the cross-domain generalization ability of the model is verified.

**Table 6:** Model performance comparison on different dataset.

Settings	Params(M)	FLOPs(G)	mIoU(%)	FPS	mDice (%)
Unet	17.30	30.68	61.80	508.87	76.10
DeepLabV3+	39.60	27.45	69.70	178.57	80.15
SwinUnet	27.15	5.92	74.67	78.79	82.35
RIFT	1.6	0.63	71.23	157.89	73.78
LMM	0.82	2.07	61.48	45.76	72.44
Feather SFASwinUnet	27.35	6.01	80.25	82.37	87.78

### 3.7 Cross-validation

To further evaluate the generalization ability and robustness of the proposed Feather SFASwinUnet model, a five-fold cross-validation experiment is performed on the SDNET2018 dataset. Specifically, the entire dataset is randomly split into five equal subsets. In each fold, four subsets are used for model training and the remaining one for validation. This process is repeated five times, with each subset serving as the validation set exactly once. The final performance of the model is determined by the average of the five validation results.

The advantage of five-fold cross-validation lies in its full utilization of all available samples for both training and validation, which effectively reduces the bias caused by a single data split and provides more reliable performance estimates. Consistent performance across all folds demonstrates the model’s favorable generalization to unseen data, while significant performance fluctuations indicate high sensitivity to data partitioning.

Table 7 presents the five-fold cross-validation results. The model achieves consistent performance across all folds, with mIoU ranging from 80.47% to 81.12% and mDice ranging from 88.05% to 88.34%. The average mIoU and mDice are 80.74% and 88.16%, respectively, with standard deviations of only 0.26% and 0.11%, demonstrating excellent stability and robust generalization capability without overfitting.

**Table 7:** The results of the cross-validation.

Fold	Training Set	Validation Set	mIoU(%)	mDice (%)
1	Fold 2,3,4,5	Fold 1	80.75	88.15
2	Fold 1,3,4,5	Fold 2	80.55	88.10
3	Fold 1,2,4,5	Fold 3	80.84	88.19
4	Fold 1,2,3,5	Fold 4	81.12	88.34
5	Fold 1,2,3,4	Fold 5	80.47	88.05
Average			80.74	88.16

### 3.8 Crack focus detection and segmentation based on YOLO11-BD

In actual bridge inspections, cracks are often distributed in complex environments. Relying solely on segmentation methods is prone to false detections and missed detections because of noise interference. Therefore, a holistic method that can combine the advantages of detection and segmentation is needed to improve the accuracy and robustness of bridge crack identification. Given that directly performing bridge crack segmentation on complex backgrounds often leads to poor results, this study adopts a hybrid approach. This method uses the YOLO11-BD model proposed in the previous study of this study team to perform focused detection of the crack area [32]. This model is optimised based on the YOLO11 framework, which can efficiently handle bridge cracks and maintain high detection accuracy for bridge cracks in complex background situations.

**Fig 6:**
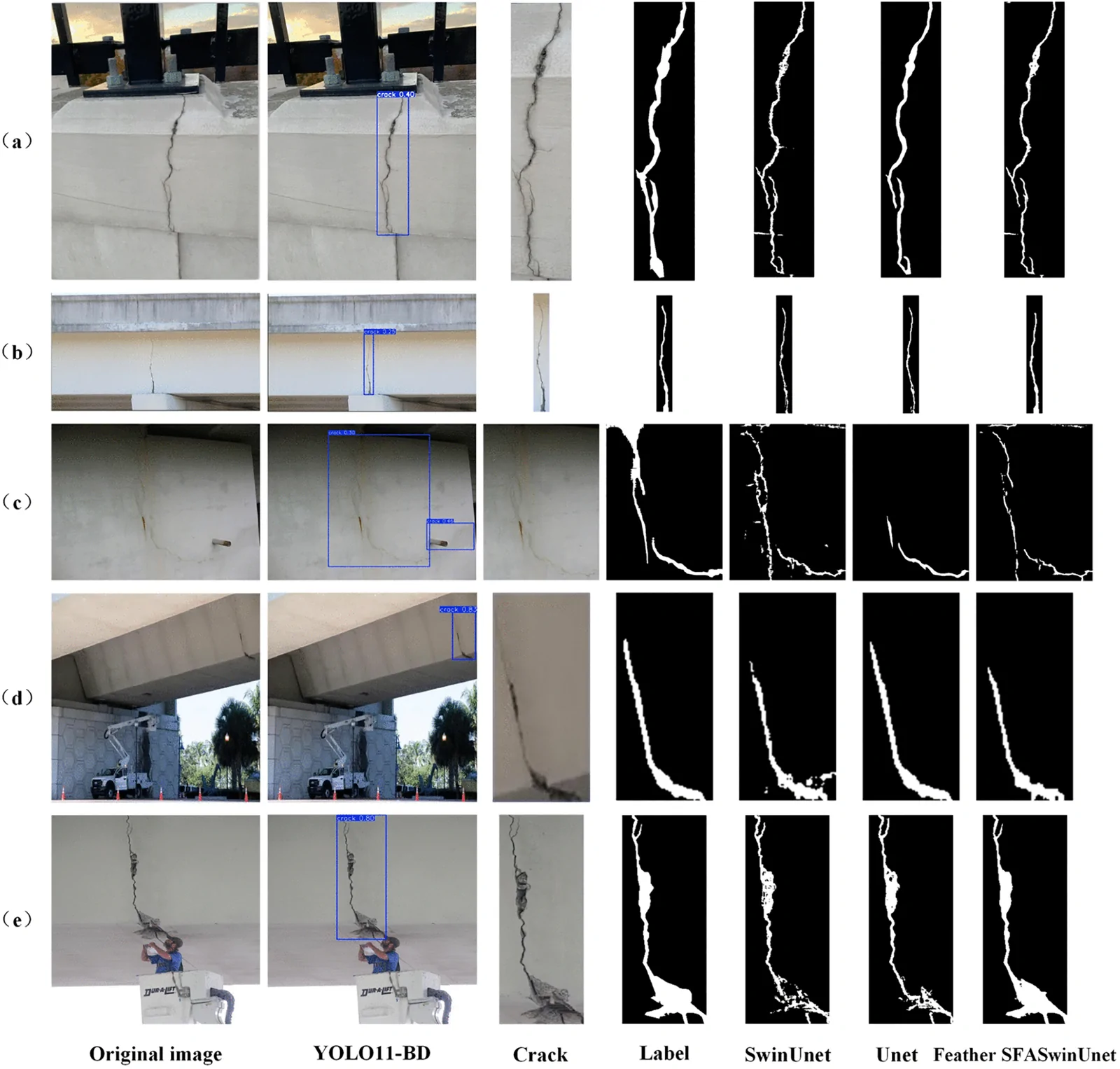
Crack segmentation results under complex background.

This part of the experiment first generates a bounding box through the YOLO11-BD model, accurately locates the crack area and then segments the image in this area. The goal of the first stage is to separate the cracks from the complex background, which makes subsequent segmentation tasks more focused and accurate. When performing crack segmentation in the second stage, only these target areas need to be processed. This way avoids the need to process the entire image, which greatly improves computational efficiency. This two-stage strategy is more effective than the traditional single-stage segmentation method, especially when the background is complex and the cracks are small. The experimental results are shown in Fig 6. Although the image background contains textures, shadows, stains and other structural interference factors on the bridge, after crack detection and positioning based on the YOLO11-BD model, the segmentation model accurately identifies and segments the crack area. For larger cracks, the model can successfully segment the complete crack area, and the segmentation effect is equally good for small cracks or parts with blurred crack edges. The outline of the crack remains relatively intact, and no obvious missed or wrong detections are observed. The experimental results fully prove the effectiveness and accuracy of the proposed hybrid detection segmentation method in segmenting bridge cracks in complex backgrounds. It effectively solves the problems of mis-identification and mis-segmentation that are easily caused by traditional segmentation methods when dealing with complex backgrounds.

It should be noted that the proposed two-stage framework inherently relies on the reliability of the first-stage detector. In cases where the YOLO11-BD model fails to detect cracks due to extremely narrow crack widths, severe occlusion, low contrast, or complex lighting conditions, the subsequent Feather SFASwinUnet segmentation model cannot recover the undetected crack in the second stage. Although such detection failures only occur in a relatively small proportion of test samples, they remain an inevitable limitation of the current pipeline. Future work will explore end-to-end detection-segmentation frameworks, including Mask R-CNN or YOLO-based instance segmentation variants, and incorporate adaptive confidence-based fallback mechanisms to mitigate error propagation.

### 3.9 Crack quantification

**Fig 7:**
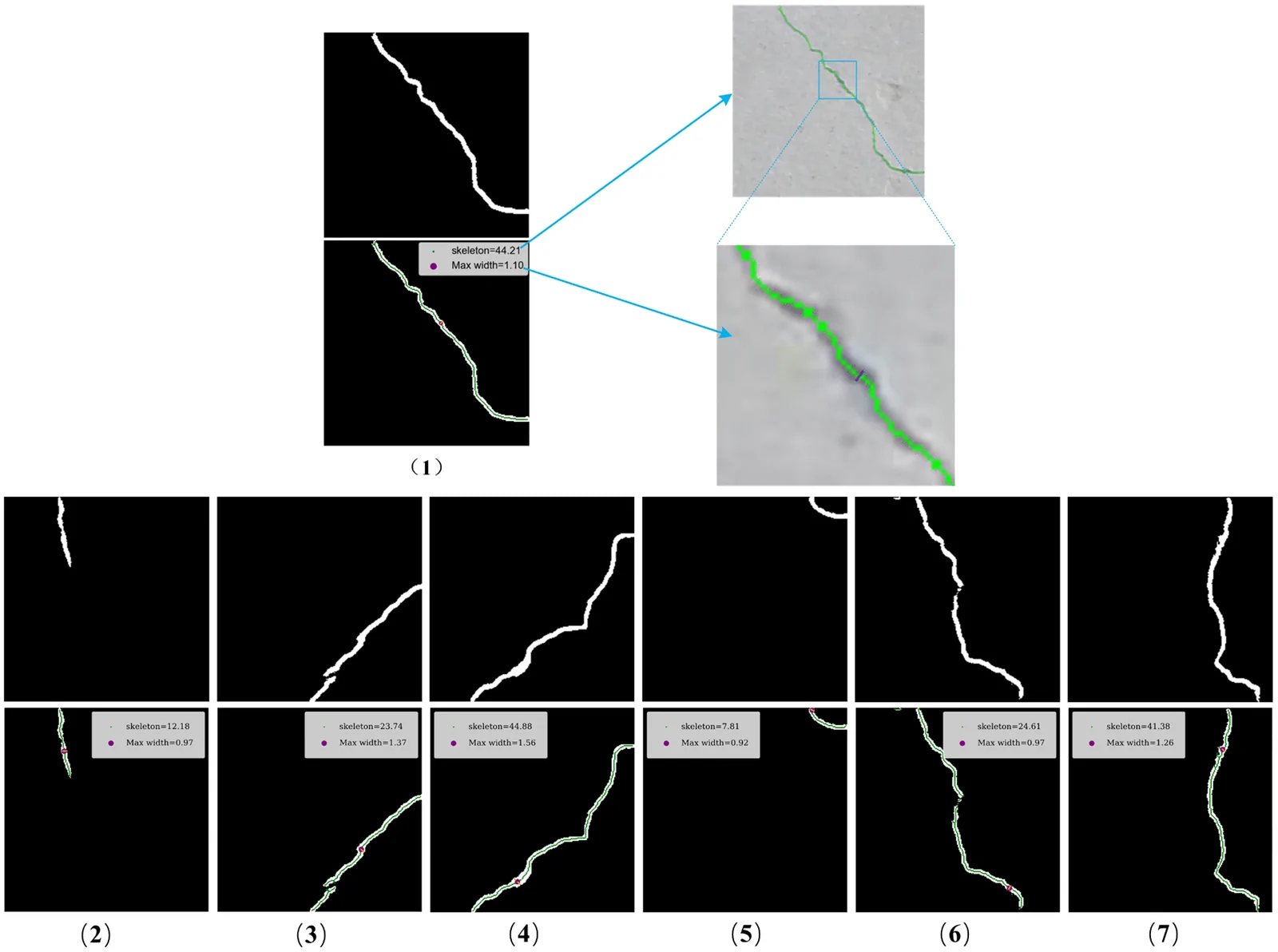
Length and width of the crack segmentation images.

To a certain extent, the single identification of bridge cracks cannot intuitively assess the health of the bridge. The width and length of the cracks can be used as key quantitative indicators. This study uses the orthogonal skeleton method to conduct a more accurate assessment of the structural characteristics of cracks. The method calculates the width and length of the crack segmentation images obtained through the Feather SFASwinUnet model. To verify the accuracy of the orthogonal skeleton method, this study randomly selects 20 model segmentation images for comparison experiments. The selected crack images contain different types of shapes and sizes to ensure the rigor of the experiment. Fig 7 shows the results of crack length and width calculated by the orthogonal skeleton method. In all selected images, the skeleton line can completely cover the main area of the crack, and the width and length of the crack have been effectively quantified. To further verify the accuracy of crack quantification, the calculated crack width is compared with the actual width measured manually. The results are shown in Table 8. Certain errors are inevitable during the crack quantification process. The main sources include edge pixelation, local blur and discontinuity at the crack edge during image segmentation, the limitation of image resolution on the expression of fine crack size, and the subjective bias introduced during manual measurement. Overall, the mean relative error across the 20 samples is 3.20% with a standard deviation of 1.18%, and all samples have errors within 5%, which indicates that crack quantification through segmentation results combined with the orthogonal skeleton method can achieve consistent and accurate measurement.

**Table 8:** Comparison of crack width calculation errors.

Crack NO.	Calculated width(px)	Actual Width(px)	Error(px)	Relative Error (%)
1	1.10	1.05	0.05	4.76
2	0.92	0.90	0.02	2.22
3	1.56	1.50	0.06	4.00
4	0.97	1.00	0.03	3.00
5	1.37	1.35	0.02	1.48
6	1.78	1.70	0.08	4.70
7	1.26	1.20	0.06	5.00
8	1.84	1.90	0.06	3.15
9	1.10	1.15	0.05	4.34
10	0.97	0.95	0.02	2.10
11	1.53	1.55	0.02	1.29
12	1.22	1.20	0.02	1.17
13	1.37	1.40	0.03	2.14
14	0.87	0.90	0.03	3.45
15	1.96	1.90	0.06	3.16
16	1.33	1.28	0.05	3.90
17	1.88	1.80	0.08	4.44
18	1.33	1.29	0.04	3.10
19	1.68	1.62	0.04	0.24
20	1.45	1.39	0.06	4.31

Given that cracks often show bends, bifurcations and irregular directions in actual structures, the measurement of crack length is more complicated than the width. As shown in the segmented image results in Fig 7, the skeleton curve can completely cover the main area of the crack, which ensures the continuity and integrity of the spatial distribution of the crack. Compared with methods that rely only on edge detection, the orthogonal skeleton method can provide stable length estimation while maintaining the overall geometric shape by extracting the centreline of the crack. The aforementioned experimental results have verified that the method has high accuracy in measuring crack width. Therefore, the orthogonal skeleton method can also be used to calculate the length of the crack. As shown in Fig 7, the crack lengths are 44.21 (px), 7.81 (px), 44.88 (px), 12.18 (px) and 23.74 (px), 24.61 (px), 41.38 (px). This study uses a quantitative calculation method that integrates crack segmentation results with the orthogonal skeleton method to systematically extract main parameters such as crack length, width and direction. This work provides more comprehensive, accurate and reliable technical support for quantitative analysis of bridge cracks. It also helps improve the scientific nature of structural safety assessment.

**Fig 8:**
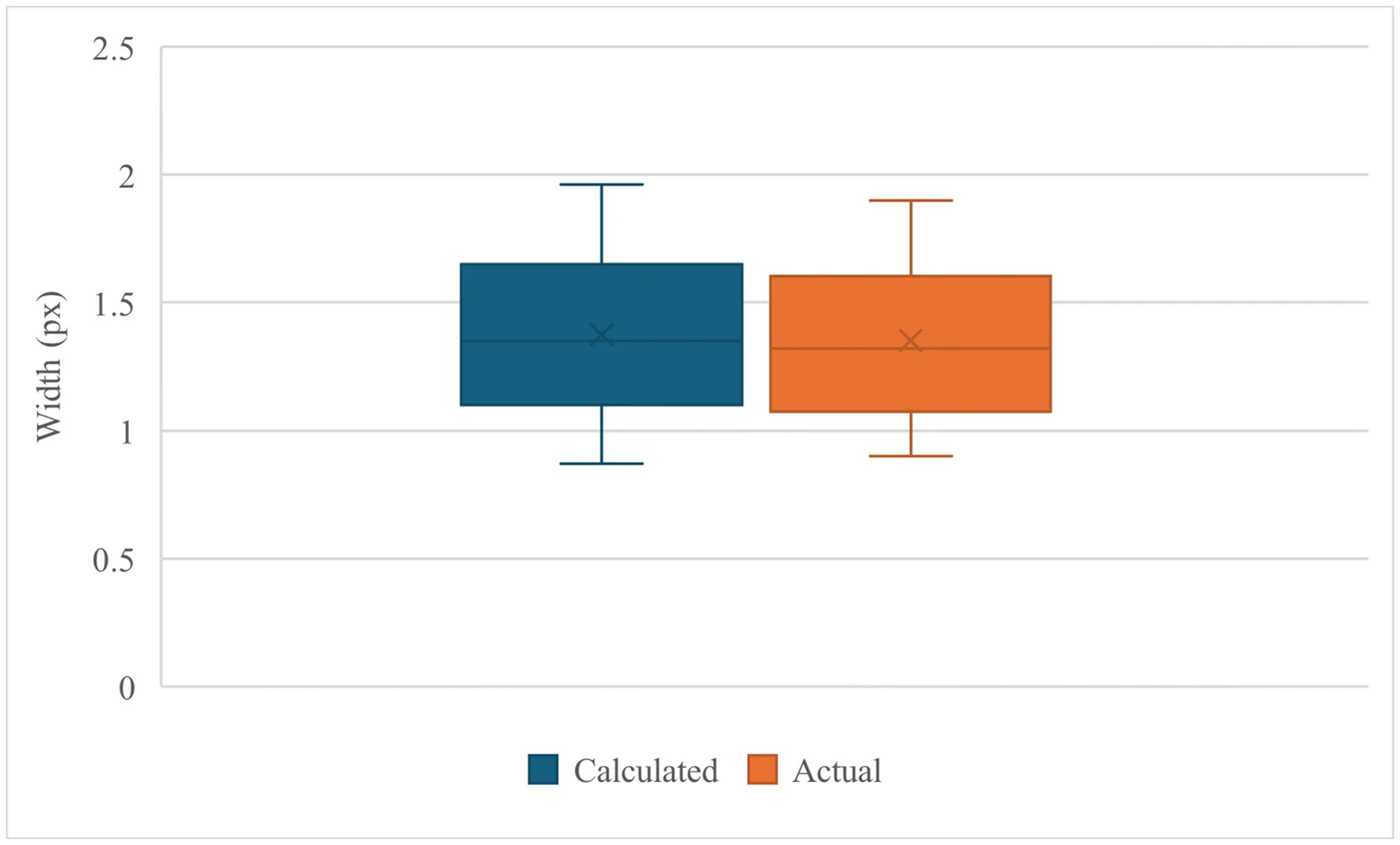
Comparison between calculated and actual crack widths.

Fig 8 presents a boxplot comparison between the calculated and actual crack widths across the 20 randomly selected samples. The two boxes exhibit nearly identical medians, interquartile ranges, and overall data distributions, indicating that the proposed quantification method based on the orthogonal skeleton can accurately estimate crack widths with negligible deviation. This excellent consistency further verifies the high accuracy and reliability of the proposed quantification method.

## 4 Conclusion

Bridge cracks are a common structural disease. Efficient and accurate intelligent detection and quantitative analysis are important to the maintenance of bridges throughout their life cycle. This study proposes the Feather SFASwinUnet, which is a bridge crack segmentation network. By introducing two methods, SFA and FCB, it achieves efficient fusion of global context and local details. The complementarity and effectiveness of SFA and FCB in the crack segmentation are verified through ablation experiments. Experimental results on the SDNET2018 dataset show that this model is significantly better than other typical models in terms of segmentation accuracy (mIoU 81.25%, mDice 88.57%). It also maintains rational computational costs and shows excellent robustness. In addition, this study combines the YOLO11-BD algorithm proposed in our previous study to achieve the detection and segmentation of bridge cracks under complex backgrounds. Lastly, in terms of crack quantification, the orthogonal skeleton method is used to extract geometric features from the model segmentation results, which verifies its accuracy in crack parameterisation measurement and accurately calculating the width and length of the crack. This study not only proposes a crack segmentation framework that combines high precision and lightweight but also provides a systematic solution for bridge health monitoring through the complete process of detection–segmentation–quantification. The subsequent study will expand the application of this method in larger-scale UAV inspection data and multi-type structural disease identification. Its deployment and promotion potential in bridge life cycle maintenance will also be explored.
